# Hospital-recorded chronic health conditions in children with and without Down syndrome in England: a national cohort of births from 2003 to 2019

**DOI:** 10.1136/archdischild-2024-327532

**Published:** 2024-10-30

**Authors:** Julia Shumway, Jill Ellis, Alice Stephens, Bianca Lucia De Stavola, Ruth Gilbert, Ania Zylbersztejn

**Affiliations:** 1 Population, Policy and Practice, University College London Great Ormond Street Institute of Child Health, London, UK; 2 East London NHS Foundation Trust, London, UK; 3 NIHR Great Ormond Street Hospital Biomedical Research Centre, London, UK

**Keywords:** Adolescent Health, Child Health, Epidemiology, Health services research, Paediatrics

## Abstract

**Objective:**

The objective is to describe age-specific cumulative incidence for hospital-recorded indicators of chronic health conditions (CHCs) in children with Down syndrome (DS) compared with children without DS.

**Design:**

National birth cohort using hospital admission and death records.

**Setting:**

National Health Service (NHS)-funded hospitals in England.

**Population:**

Liveborn, singleton infants born in NHS-funded hospitals between 2003 and 2019.

**Main outcome measures:**

Cumulative incidence of nine categories of hospital-recorded CHCs, multimorbidity and mortality.

**Results:**

We identified 10 621 infants with DS among 9 631 646 liveborn, singleton infants (0.11%). Among children with DS, the cumulative incidence for any indicated CHC was 90.1% by age 16, as compared with 21.2% of children without DS. By age 16, a third of children (33.1%) with DS had CHCs affecting four or more body systems; only 6.0% of children without DS had CHCs indicated in more than one body system. The most common CHCs in children with DS were severe congenital heart defects, indicated in 57.2% (0.8% in children without DS). The estimated HR for mortality up to age 16 comparing children with versus without DS was 15.26 (95% CI: 14.15, 16.45).

**Conclusions:**

Children with DS had a higher cumulative incidence for CHCs in each body system category and subcategory, at all ages, than children without DS. Multimorbidity and mortality were higher among children with DS. Administrative data can be used to examine the health needs and healthcare use of children with DS throughout childhood and adolescence.

**WHAT IS ALREADY KNOWN ON THIS TOPIC**

- Children with Down syndrome (DS) have an increased risk for a variety of chronic health conditions (CHCs); routine health assessments improve the timeliness of treatment.

**WHAT THIS STUDY ADDS**

- Children with DS have a higher cumulative incidence of CHCs at all ages and across all body systems compared with their peers without DS.
- Not only do nearly all (90.1%) children with DS have a hospital-recorded CHC by age 16, but the majority also have conditions recorded in two or more body systems.

**HOW THIS STUDY MIGHT AFFECT RESEARCH, PRACTICE OR POLICY**

- Because of their frequent interactions with secondary healthcare, hospital admission data are a valuable resource for studying CHCs in children with DS.
- Hospital Episode Statistics provide a consistent record of children with DS’s long-term healthcare use which can linked to other data sources to study the implications of complex healthcare needs for educational, social and other outcomes.

## Introduction

 Down syndrome (DS) is the most common chromosomal disorder, affecting 1 in every 873 (0.12%) liveborn infants in 2020 England.^1^ Children with DS have increased risk for chronic health conditions (CHCs), including congenital heart disease (CHD), hypothyroidism, epilepsy, leukaemia and sleep apnoea.^2^,^4^ Improved treatment for these conditions has contributed to an increased average life expectancy for people with DS, from 12 years in the 1950s to approximately 60 years today.^5^

National guidelines recommend regular assessments for children with DS to detect and respond to CHCs.^6^ Appropriately timed surveillance requires an understanding of the risk for CHCs by age.^7^ Recent DS research has presented incidence rates for CHCs in adult populations and period prevalences for CHCs in children, but evidence is lacking regarding the age at recording for CHCs throughout childhood.^28^,^10^

We used a national cohort of children born in England from 2003 to 2019 linked to hospital admission and mortality records to describe the age-specific cumulative incidence of hospital-recorded CHCs among children with DS from birth through 16 years, in comparison with contemporary peers without DS. We reported cumulative incidences of CHCs (overall and by type), multimorbidity and mortality.

## Methods

### Data sources

We used Hospital Episode Statistics (HES) Admitted Patient Care data linked with Office for National Statistics (ONS) death records from 1 January 2003 to 1 January 2020. HES contains administrative and demographic information for all hospital admissions funded by the National Health Service (NHS) (99% of all English hospital activity) since 1997.^11^ Each record includes up to 20 diagnoses recorded using codes from the International Classification of Diseases, Tenth Revision (ICD-10 codes).^12^ ONS records provide up to 15 contributing causes for deaths registered in England and Wales.^13^

### Study population

We derived a national cohort of singleton, liveborn infants delivered in English hospitals (capturing 97% of liveborn, singleton infants) from 1 January 2003 to 1 January 2019 using previously described methods.^14 15^ We excluded multiple births because they link less successfully to subsequent hospital admissions.^14^ We followed children until age 16, death or 1 January 2020, whichever came first. Stopping follow-up on 1 January 2020 allowed a full year of follow-up for all cohort members prior to reductions in hospital admissions caused by the COVID-19 pandemic.^16^

We divided the cohort into two groups: children with DS and children without DS. Children with DS were identified by an ICD-10 code of Q90 recorded in any hospital admission or death registration, regardless of the age when recorded. Children without DS never had a Q90 code recorded.

### Chronic health conditions

We identified CHCs using an adapted Hardelid code list for chronic conditions likely to require at least one year of follow-up care.^17^ We revised included codes and groupings to reflect conditions relevant to children with DS based on guidelines published by the DS Medical Interest Group,^7^ previous research^10 18^ and consultation with two paediatricians (JE and RG; online supplemental material).

We grouped CHCs into nine body system categories:

1. Autoimmune, endocrine and metabolic
2. Cancers and blood disorders.
3. Cardiovascular.
4. Central nervous system (CNS).
5. Digestive, renal and genitourinary (GU).
6. Developmental and behavioural
7. Musculoskeletal.
8. Respiratory.
9. Vision and hearing.

We used codes indicating the presence of a CHC without providing detail necessary to group into any specific body system (eg, wheelchair use and palliative care) to calculate the incidence for having any CHC, but not for indicating multimorbidity or CHCs affecting specific body systems.

Within each body system category, we grouped ICD-10 codes into specific subcategories relevant to children with DS (eg, severe CHD, hypothyroidism), and we classified body system-specific conditions present from birth as congenital anomalies.

We defined *incidence date* as the first date a relevant ICD-10 code was recorded as any diagnosis or cause of death in HES or ONS records. When a child had multiple ICD-10 codes indicating the same body system, we used the date of the first as the incidence date for that body system. For example, when a child’s records indicated both asthma and sleep apnoea, only the first recorded code counted towards the cumulative incidence of a CHC in the respiratory system category.

### Cohort characteristics

We collated characteristics recorded in birth admissions and supplemented missing data on birth weight, gestational age and maternal age using maternal delivery records.^14 15 19^ Cohort characteristics at birth were sex, ethnicity category (white, Asian/Asian British, black/black British, mixed and other), calendar year of birth (in 4-year groups), birth weight (<1500 g, 1500–2499 g, ≥2500 g), gestational age in weeks (<32, 32–36, ≥37), maternal age in years (<20, 20–24, 25–29, 30–34, 35–39, ≥40) and a quintile of neighbourhood deprivation indicator, the Index of Multiple Deprivation (IMD). The IMD ranks neighbourhoods (averaging 650 households) based on a composite of domains, including income deprivation, crime and unemployment.^20^

### Statistical analysis

We estimated the prevalence of children with DS within the cohort. We described cohort characteristics at birth by DS status.

We performed parallel analyses for the groups of children with and without DS, estimating the cumulative incidence for the first indicator of any CHC (including unclassified) and the cumulative incidences for CHCs in each body system category and subcategory using the Aalen-Johansen method. In addition to accounting for variable follow-up time, the Aalen-Johansen method addresses potential bias resulting from deaths associated with other CHCs by differentiating between death and other causes of loss-to-follow-up (eg, end of study period).^21 22^ We reported all cumulative incidences by ages 1, 5, 11 and 16 years, by DS group.

We estimated mortality incidence by 16 years for both groups using the Kaplan-Meier method, and we estimated the HR comparing the groups by fitting a Cox proportional hazards regression model.^23^

We conducted all analyses using the survival package in R-Studio (V.4.3.0).^23^

### Sensitivity analyses

We investigated changes in diagnostic coding depth over time by plotting cumulative incidence curves for first-recorded CHC by DS group and birth year and by calculating the annual average number of recorded ICD-10 codes per hospital record among children with DS.

## Results

**Figure 1:**
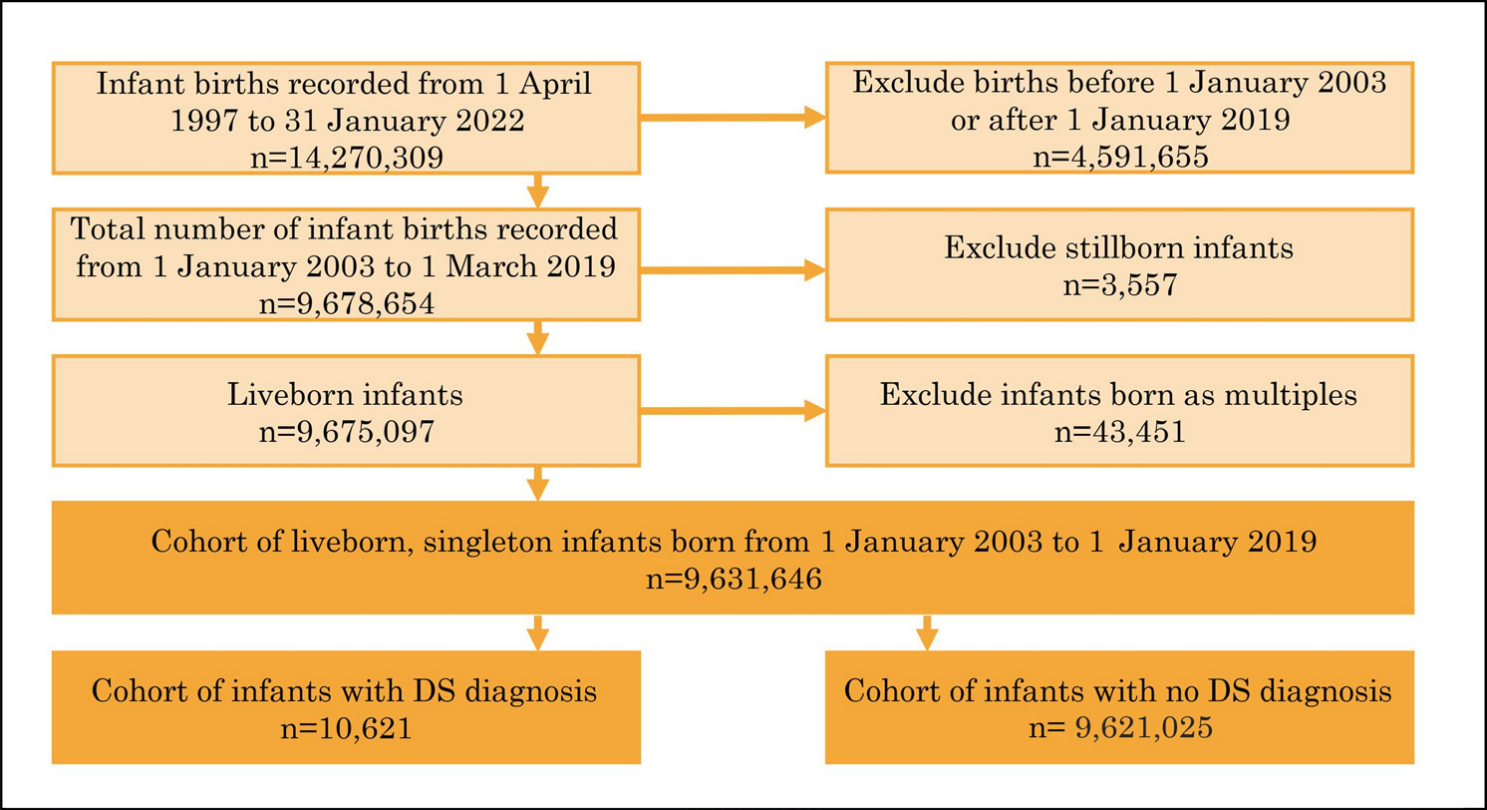
Infants born in English hospitals from 1 January 2003 to 1 January 2019. DS, Down syndrome.

We identified 9 631 646 liveborn, singleton infants delivered in NHS hospitals from 1 January 2003 to 1 January 2019, including 10 621 (0.11%) whose records indicated DS and 9 621 025 whose did not indicate DS (figure 1). This is consistent with surveillance reports for England.^1^

### Cohort characteristics

Sex, ethnicity, birth year and IMD were similarly distributed between children with and without DS (table 1). More children with DS were born weighing under 2500 g (20% compared with 5%), before 37 weeks’ gestation (19% compared with 5%), and to mothers 35 years and older (44% compared with 17%). More children without DS had missing data for ethnicity and deprivation status, but less missing data on birth characteristics.

**Table 1:** Characteristics of the cohort of liveborn, singleton infants born in English hospitals between 1 January 2003 and 1 January 2019, grouped by Down syndrome (DS) status

Characteristics at birth	DS group	Non-DS group
	n (%)	
All	10 621 (100%)	96 210 125 (100%)
Sex		
Male	5460 (51%)	4 745 180 (49%)
Female	4532 (43%)	4 514 788 (47%)
Missing	629 (6%)	361 057 (4%)
Ethnicity		
White	6623 (62%)	6 188 957 (64%)
Asian or Asian British	1203 (11%)	918 876 (10%)
Black or black British	855 (8%)	446 473 (5%)
Mixed	465 (4%)	376 451 (4%)
Other	305 (3%)	222 940 (2%)
Missing or unknown	1170 (11%)	1 467 328 (15%)
Birth year		
2003–2006	2482 (23%)	2 231 327 (23%)
2007–2010	2651 (25%)	2 462 815 (26%)
2011–2014	2835 (27%)	2 519 205 (26%)
2015–2019	2653 (25%)	2 407 678 (25%)
Birth weight		
<1500 g	299 (3%)	63 562 (1%)
1500–2499 g	1857 (17%)	404 174 (4%)
≥2500 g	6896 (65%)	7 928 629 (82%)
Missing	1569 (15%)	1 224 660 (13%)
Gestational age		
<32 weeks	266 (3%)	66 318 (1%)
32–36 weeks	1730 (16%)	389 204 (4%)
≥37 weeks	6575 (62%)	7 537 815 (78%)
Missing	2050 (19%)	1 627 688 (17%)
Maternal age		
<20 years	235 (2%)	426 338 (4%)
20–24 years	792 (7%)	1 471 724 (15%)
25–29 years	1220 (11%)	2 275 341 (24%)
30–34 years	1997 (19%)	2 447 449 (25%)
35–39 years	2752 (26%)	1 353 956 (14%)
≥40 years	1939 (18%)	308 693 (3%)
Missing	1686 (16%)	1 337 524 (14%)
Index of Multiple Deprivation Quintiles		
Most deprived 20%	3188 (30%)	2 603 561 (27%)
Most deprived 20%–40%	2368 (22%)	2 069 232 (22%)
Middle 20%	1823 (17%)	1 720 027 (18%)
Least deprived 20%–40%	1604 (15%)	1 513 069 (16%)
Least deprived 20%	1518 (14%)	1 533 794 (16%)
Missing or unknown	120 (1%)	181 342 (2%)

### Cumulative incidence of CHCs and multimorbidity

The cumulative incidence for any hospital-recorded CHC among children with DS was 64.6% by age 1 and 90.1% by age 16. Among children without DS, these figures were 6.1% and 21.2%, respectively (online supplemental material).

**Figure 2:**
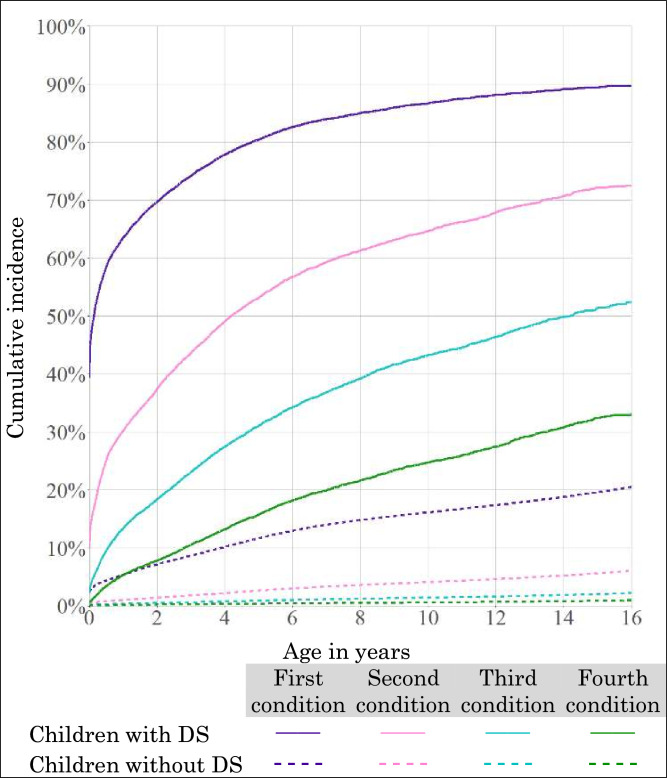
Cumulative incidence of multimorbidity based on indications for chronic health conditions affecting multiple body system categories, by Down syndrome (DS) status.

Multimorbidity was common in children with DS: nearly two-thirds (63.6%) had a CHC affecting one body system indicated by age 1; two-thirds (66.1%) had CHCs in two by age 11 and a third (33.1%) had CHCs affecting four or more body systems by age 16. In contrast, only 5.4% of children without DS had a CHC in one body system indicated by age 1, and only 6.0% had CHCs in two body systems by age 16 (figure 2).

### Cumulative incidence of CHCs by body system

**Figure 3:**
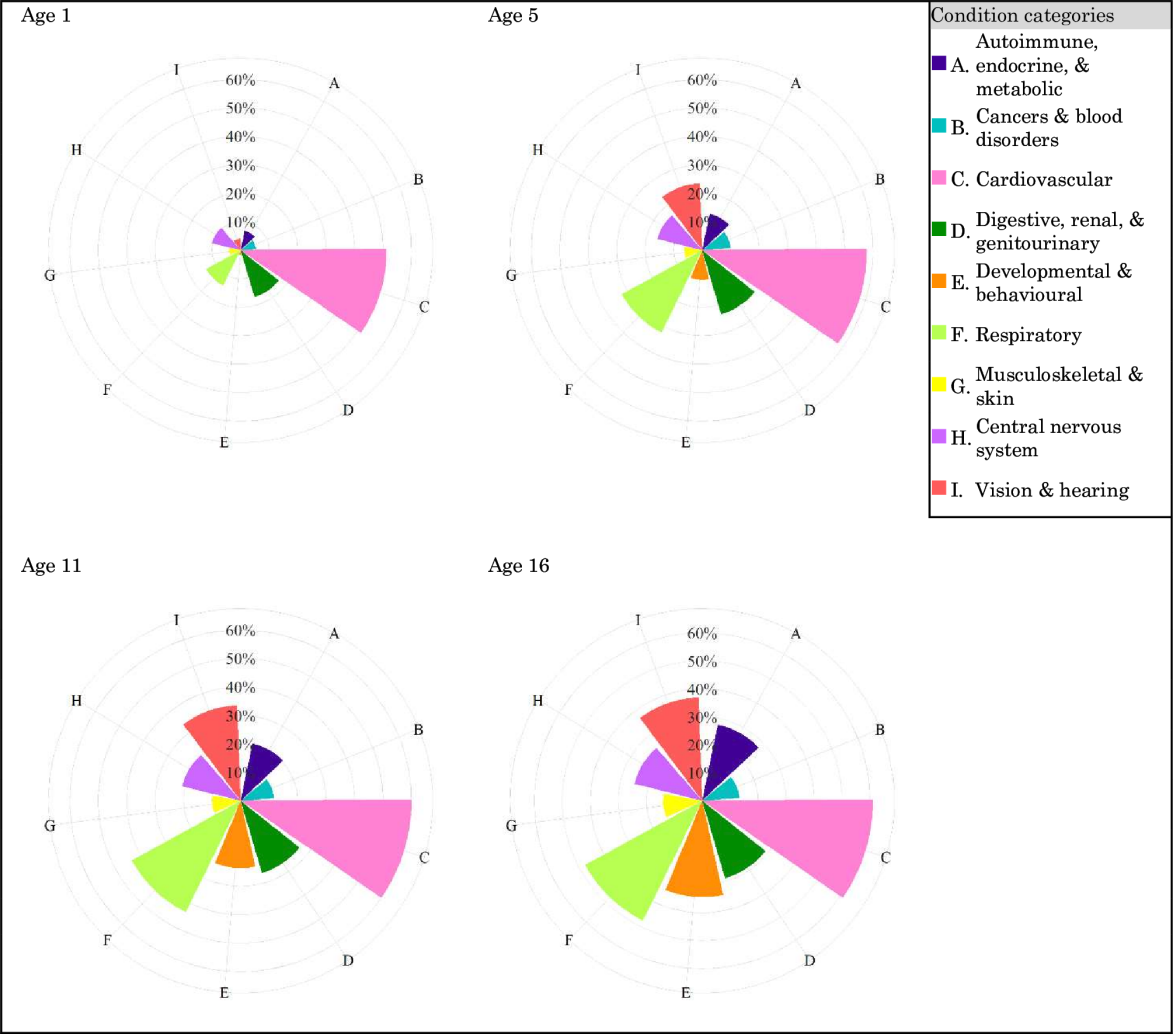
The cumulative incidence of chronic health conditions among children with Down syndrome.

The cumulative incidence of CHCs was higher among children with DS for each body system category and subcategory. Among children with DS, CHCs in the cardiovascular system had the highest cumulative incidence by age 16 (61.1%), followed in descending order by respiratory (47.7%); vision and hearing (37.0%); developmental and behavioural (34.4%); digestive, renal and GU (28.9%); autoimmune, endocrine and metabolic (27.8%); CNS (25.0%); musculoskeletal and skin (14.0%); and cancers and blood disorders (13.4%) (figure 3 and online supplemental material).

### Subcategories

**Figure 4:**
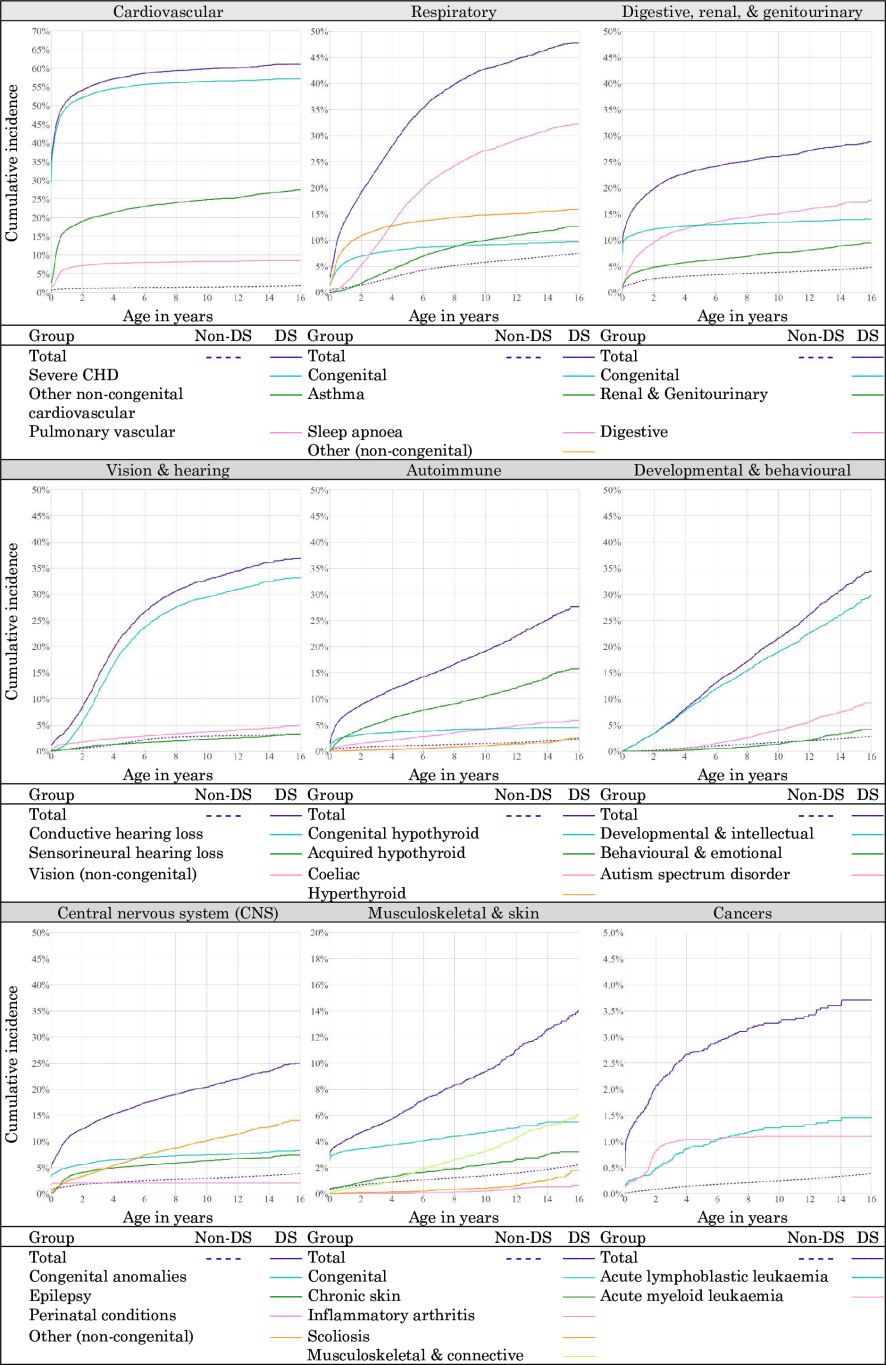
Cumulative incidence for chronic health conditions, by body system categories and selected subcategories, among children with and without Down syndrome (DS). Note: The vertical axes for cardiovascular, musculoskeletal and skin, and cancers are on different scales to the other plots. CHD, congenital heart disease.

A majority of children with DS had hospital-recorded severe CHD (57.2%), and by age 16, 29.8% had a non-congenital cardiovascular condition. Most of these were recorded subsequent to an indication of severe CHD. In children without DS, severe CHD and non-congenital cardiovascular conditions (eg, pulmonary vascular disease) were rare (0.8% and 1.3% by age 16, respectively) (figure 4).

The most common subcategories of respiratory CHCs among children with DS by age 16 were sleep apnoea (32.2%) and asthma (12.6%); among children without DS; however, asthma was more common (5.3%) than sleep apnoea (1.4%).

Conductive hearing loss was the most recorded subcategory of vision and hearing CHCs, with hospital-recorded cumulative incidence increasing steeply during early childhood (20.5% by age 5, compared with 1.3% for children without DS).

More than one-third (34.4%) of children with DS had a comorbid developmental or behavioural condition indicated by age 16, compared with 2.8% of children without DS. The cumulative incidence of hospital-recorded autism spectrum disorder (autism) was 9.2%, more than seven times higher than that of children without DS (1.3%).

Congenital digestive, renal and GU conditions were indicated for 14.0% of children with DS, but this cumulative incidence was surpassed by non-congenital conditions by age 5 (16.4%). Corresponding figures for children without DS were 1.3% and 2.3%.

Acquired hypothyroidism was the most common autoimmune condition among children with DS (15.8% by age 16). Other metabolic and endocrine conditions affected 10.0% of children with DS by age 16; compared with DS were 0.1% and 1.5% for children without DS.

‘Other’ neurological conditions were the most recorded CNS conditions among children with DS (14.0%), followed by epilepsy (7.4%). In children without DS, these figures were 1.8% and 1.5%. Most cases of epilepsy were first recorded by age 5 (DS group 5.2%, non-DS group: 0.9%).

Indications of musculoskeletal and skin conditions among children with DS increased during adolescence: the cumulative incidence of musculoskeletal and connective conditions was 1.5% by age 5 and 6.1% by age 16; scoliosis was 0.2% by age 5 and 1.8% by age 16. The corresponding figures by age 16 for children without DS were 1.0% and 0.1%.

Cancer was indicated in 3.7% of children with DS by age 16 and exhibited the distinct pattern expected for acute myeloid leukaemia (AML): increasing from 0.3% by age 1 to 1.1% by age 16, with a steep incline just before age 2 (figure 4).^24^ In contrast, the cumulative incidence for cancer in children without DS was 0.4% by age 16, with AML recorded infinitesimally rarely (0.0%).

### Mortality

The Kaplan-Meier cumulative incidence of death was 7.0% among children with DS versus 0.5% among children without DS, by age 16, with an estimated mortality HR comparing DS versus non-DS of 15.26 (95% CI: 14.15, 16.45) (online supplemental material).

### Sensitivity analyses

The cumulative incidence for first recorded CHC was higher at younger ages for children born in later years (online supplemental material). This may be due to an increase in the average number of ICD-10 codes recorded for each hospital episode over time (online supplemental material).

## Discussion

### Key findings

Within a nationally representative cohort of 9 631 646 liveborn, singleton infants born in England between 2003 and 2019, we identified 10 621 children with DS. 90.1% of children with DS had hospital-recorded CHCs by age 16, and 72.5% had CHCs recorded for two or more body systems. Children with DS had a higher cumulative incidence of CHCs affecting each body system and system subcategory, compared with children without DS. Cardiovascular conditions and severe CHD were the body system and subcategory with the highest cumulative incidence for children with DS.

### Strengths and weaknesses

HES provided a nearly universal cohort, representing 97% of English liveborn, singleton infants. Following this representative cohort longitudinally allowed us to estimate the cumulative incidences of rare CHCs indicated in hospital admission or death records.^11^ We provided a comprehensive overview of CHCs affecting all body systems, and presented subcategories relevant to children with DS. Our methods provide a model for exploring more specific conditions in future studies.

Our results most accurately reflect CHCs treated and recorded during hospital admissions and may underestimate and reflect a delay in recording conditions diagnosed and treated in primary care, mental health, and community services, which are not included in HES. Such CHCs relevant to DS include autism,^25^ asthma^26^ and conductive hearing loss.^27^ Some CHCs recorded during hospital admissions may represent complications from conditions previously managed in outpatient settings or whose hospital management was recorded using treatment rather than diagnostic codes. Children with complex healthcare needs are more likely to have their comorbidities recorded, as a high frequency of hospital visits provides more ‘opportunity’ for recording.^28^ Additionally, certain CHCs recognised during the frequent routine standard care of children with DS may be considered relevant to managing their hospital care.^29^ For example, asthma and sleep apnoea may be recorded as relevant to managing respiratory care for a child who receives anaesthesia during a hospital admission, whereas conductive deafness may not. While cumulative incidence estimates using HES underestimate the incidence seen in the wider healthcare system, this underestimation may vary by condition, and morbidity in children with DS is more likely to be captured than for their unaffected peers.

We showed that hospital recording of CHCs increased over time across birth years, with the sharpest increases in later birth years, likely reflecting the increase in average number of ICD-10 codes over time. Coding depth has increased since the introduction of ‘payment by results’ in 2004, which gives hospitals financial incentives to include more diagnostic codes include in each hospital record,^30 31^ and the number of possible codes per record increased from 14 to 20 in 2007.^11 30^ Additionally, the rate of admission for children with DS has increased over time,^32^ so recording may be more complete and timely for children born in later years of the cohort.

### Comparison with other studies

We compared the cumulative incidences for CHCs among children with DS against two recent studies of CHCs in children with DS (online supplemental material). McKenna’s research supplemented HES and ONS with data from the National Cancer Registration and Analysis Service to report similar prevalences for several conditions likely to be treated in a hospital setting (eg, leukaemia, congenital cardiovascular conditions, epilepsy), but may have reported values closer to the true prevalence for conditions likely to be treated in community settings, such as autism. Fitzgerald’s results for certain conditions, based on Western Australian hospital records, may indicate differences in treatment protocols (eg, 47.9% prevalence for otitis media vs this paper’s cumulative incidence of 33.3%).

### Implications

Children with DS have a higher risk for developing CHCs across all body systems than their peers. Many children with DS have congenital anomalies, and more than half (57.2%) are born with a severe CHD. Clinicians, parents and other carers can inform their surveillance and care by understanding the risks for CHCs affecting children with DS.

Hospital databases lend themselves to researching conditions likely to be seen in hospital settings. HES is particularly useful for longitudinal research and studying rare conditions, such as congenital anomalies in small populations, because it includes standardised coding for all conditions recorded during hospital admissions in England across several decades. By providing a relatively consistent record of children’s long-term healthcare use, the HES database provides a basis for exploring the health implications of their access to other services, such as education and social care, through linked administrative data.^33^

## supplementary material

10.1136/archdischild-2024-327532 online supplemental file 1

## Data Availability

Data may be obtained from a third party and are not publicly available.
