# Supplementary material for: Hospital-recorded chronic health conditions in children with and without Down syndrome in England: a national cohort of births from 2003 to 2019
Source: Arch Dis Child. 2024 Oct 30;110(3):e327532. doi: 10.1136/archdischild-2024-327532 (PMC11866298; doi:10.1136/archdischild-2024-327532)
Supplement: online supplemental file 1 [file archdischild-110-3-s001.pdf]

Supplemental material

Supplemental Table 1: Glossary of abbreviations

Glossary of abbreviations	
ASD	Atrial septal defects
Autism	Autism spectrum disorder
AVSD	Atrioventricular septal defects
AML	Acute myeloid leukaemia
CHC	Chronic health condition
CHD	Congenital heart defect
CNS	Central nervous system
DS	Down syndrome
GU	Genitourinary
HES	Hospital Episode Statistics
ICD-10	International Classification of Diseases, Tenth Revision
IMD	Index of Multiple Deprivation
ONS	Office for National Statistics
M & S	Musculoskeletal and skin
NHS	National Health Service
ToF	Tetralogy of Fallot
UT	Urinary tract
VSD	Ventricular septal defects

Supplemental Table 2: ICD-10 codes indicating chronic health conditions by body system categories and subcategories

	Mid-category	Subcategory	Codes
Autoimmune, endocrine, and metabolic	Autoimmune	Coeliac disease	K90
		Hyperthyroidism	E05, E06.3
		Congenital hypothyroidism	E03.0, E03.1
		Acquired hypothyroidism	E03.2-E03.9, Y42.1
		Other autoimmune	G73.1
	Other metabolic and endocrine conditions	Diabetes	E10-E14, G59.0, G63.2, I79.2, N08.3, O24, Y42.3
		Other endocrine and metabolic conditions	D55, E00, E07.1, E22.0, E23.0, E25, E26.8, E29.1, E31, E34.1, E34.2, E34.5, E34.8, E66, E70-E72, E74-E78, E79.1-E80.3, E80.5, E80.7, E83, E85, E88.0-E88.2, E88.8, E88.9, G13.2, G63.3, G73.5, G73.6, L99.0, N16.3, N92, Q87.8, Q89.1, Q89.2, Z86.3, Z93.8
Cancers and blood disorders	Cancers	Acute myeloid leukaemia	C92
		Acute lymphoblastic leukaemia	C90.1-C95
		Other cancers	C00-C90.0, C96, C97, D00-D02, D05-D09, D12, D13, D14.1-D14.4, D15, D20, D32-D35, D37-D48, D63.0, E34.0, E88.3, G13.0, G13.1, G53.3, G55.0, G63.1, G73.1, G73.2, G94.1, M36.0, M36.1, M82.0, M90.6, M90.7, N08.1, N16.1, Y43.1-Y43.3, Y84.2, Z08, Z51.0, Z51.1, Z54.1, Z54.2, Z85, Z86.0, Z92.3
	Blood disorders	Anaemia, immunological, and other blood disorders	D50, D56.0-D56.2, D56.4, D56.8, D56.9, D57.0-D57.2, D57.8, D58, D61.0, D61.9, D64, D66, D67, D68.0-D68.2, D68.4-D68.9, D69, D70-D76, D80-D84, G53.2, M36.2-M36.4, M90.4, N08.2, Q98.0, Z86.2
Cardiovascular	Severe congenital heart defects[1]	Ventricular septal defect	Q21.0
		Atrial septal defect	Q21.1
		Atrioventricular septal defect	Q21.2
		Tetralogy of Fallot	Q21.3
		Other severe congenital heart defects	Q20.0, Q20.1, Q20.2, Q20.3, Q20.4, Q20.5, Q20.6, Q21.4, Q21.8, Q22.0, Q22.4, Q22.5, Q22.6, Q23.0, Q23.2, Q23.4, Q24.2, Q24.4, Q24.5, Q25.1, Q25.2, Q25.3, Q26.2, Q26.3
	Non-congenital cardiovascular disease	Pulmonary vascular disease	I27, I28
		Other cardiovascular disease	I00-I26, I31-I39, I41, I42.0-I42.5, I42.7-I43.8, I44.1-I44.7, I45.1-I45.9, I46-I51, I52.8, I70-I71, I72.1-I72.4, I72.8, I72.9, I73-I77, I79.0, I79.1, I79.8, I81, I82, I98, I99, M03.6, N08.8, Q27, Q28, S26, T82.0-T82.3, T82.5-T82.9, T86.2, Y60.5, Y61.5, Y62.5, Y84.0, Z45.0, Z50.0, Z94.1, Z95
Central nervous system (CNS)	Congenital and perinatal anomalies	Congenital anomalies of the CNS	Q00-Q07, Q16, Q75.0, Q75.1, Q85, Q86.0, Q86.1, Q86.8, Q91-Q93, Q95.2, Q95.3, Q97, Q99
		Perinatal conditions	P10, P21.0, P52, P57, P90, P91.1, P91.2, P91.6
	Non-congenital neurological conditions	Epilepsy	F80.3, G40.0-G40.4, G40.6-G40.9, G41, R56.8, Y46.0-Y46.6
		Other	F02.2, F02.3,G00-G09, G10-G12, G13.8, G14, G20-G23, G24.1-G24.9, G25-G30, G31.0-G31.1, G31.8, G31.9, G32-G37, G43-G47.2, G47.4-G53.1, G53.8, G54, G55.8, G56-G58, G59.8, G60-G62.0, G62.2-G62.9, G64, G70, G71, G72.2-G73.0, G73.3, G80-G83, G90-G93, G94.2, G94.8, G95, G96, G98, G99.1, G99.2, I60-I67, I68.0, I68.2, I69, I72.0, I72.5, T85.0, T85.1, Y46.7, Y46.8, Z98.2

[1] The use of the term "severe congenital heart defects" is based on the EUROCAT Guide 1.5 category of congenital anomalies of the same name and does not distinguish between conditions' required treatments or likelihood of subsequent mortality.(1,2)

<table>
<tr><td rowspan="7">Digestive, renal, and genitourinary (GU)</td><td rowspan="5">Congenital digestive, renal, and GU anomalies</td><td>Upper gastro-intestinal anomalies</td><td>Q39, Q40.2-Q41</td></tr>
<tr><td>Lower gastro-intestinal anomalies</td><td>Q42, Q43.1</td></tr>
<tr><td>Other gastro-intestinal anomalies</td><td>Q38.0, Q38.3, Q38.4, Q38.6-Q38.8, Q43.3-Q43.7, Q43.9-Q45, Q79.0, Q79.2, Q79.3, Q79.5</td></tr>
<tr><td>Genital anomalies</td><td>Q50-Q52, Q54-Q56</td></tr>
<tr><td>Kidney and urinary tract anomalies</td><td>Q60-Q64, Q79.4</td></tr>
<tr><td rowspan="2">Non-congenital digestive, renal, and GU conditions</td><td>Digestive</td><td>K20, K21.0, K22, K23.8, K25-K28, K29.0, K29.1, K29.3-K29.9, K31, K50-K52, K55, K57, K59.2, K63.0-K63.3, K66, K72-K76, K80-K83, K85.0, K85.1, K85.8, K85.9, K86.1-K86.9, K87.0, T86.4, Z43.2-Z43.4, Z46.5, Z90.3, Z90.4, Z93.2-Z93.5</td></tr>
<tr><td>Renal and GU conditions</td><td>D63.8, G63.8, G99.8, I68.8, M90.8, N00-N05, N07, N08.6, N11-N16.0, N16.2, N16.4-N16.8, N18-N26, N28, N29, N31, N32, N33.8, N35, N36, N39.1, N39.3, N39.4, N40-N42, N70-N74, N80-N82, N85-N88, P96.0, T82.4, T83.1, T83.2, T83.4-T83.9, T85.5, T86.1, Y60.2, Y61.2, Y62.2, Y84.1, Z49, Z93.6, Z94.0, Z99.2</td></tr>
<tr><td rowspan="3">Develop-mental and behavioural</td><td colspan="2">Autism spectrum disorders</td><td>F84</td></tr>
<tr><td colspan="2">Behavioural and emotional disorders</td><td>F90-F98</td></tr>
<tr><td colspan="2">Developmental and intellectual disabilities</td><td>F70-F79, F80.0-F80.2, F80.8, F80.9, F81-F83, F88, F89,</td></tr>
<tr><td rowspan="5">Musculoskeletal and skin</td><td colspan="2">Congenital musculoskeletal and skin anomalies</td><td>Q18.8, Q65.0-Q65.2, Q65.8, Q65.9, Q67.5, Q68.2-Q68.5, Q71-Q74, Q75.3-Q75.9, Q76.1-Q76.4, Q77, Q78, Q79.6, Q79.8, Q82.0-Q82.4, Q82.9, Q86.2, Q89.7-Q89.9</td></tr>
<tr><td rowspan="4">Non-congenital musculoskeletal and skin conditions</td><td>Scoliosis</td><td>M41</td></tr>
<tr><td>Inflammatory arthritis</td><td>M05-M14.0, M14.2-M14.8</td></tr>
<tr><td>Musculoskeletal and connective tissue conditions</td><td>G55.1-G55.3, G63.5, G63.6, G73.7, J99.0, J99.1, L62.0, M30-M35, M40, M42, M43, M45-M48, M49.5, M50-M54, M60-M62, M63.8, M80.1-M80.9, M81.1-M81.9, M82.1, M82.8, M84.0-M84.2, M84.8, M84.9, M85, M86.3-M86.6, M89, M90.0, M91-M94, M49.5, N08.5, Y45.4</td></tr>
<tr><td>Chronic skin disorders</td><td>L10, L11.0, L11.8, L11.9, L12-L14, L28, L40-L45, L57, L58.1, L59, L87, L88, L90, L92, L93, L95, L98.5, Q80, Q81, Q87.0-Q87.5, Q89.4</td></tr>
<tr><td rowspan="4">Respiratory</td><td colspan="2">Congenital respiratory anomalies</td><td>Q30-Q37</td></tr>
<tr><td rowspan="3">Non-congenital respiratory conditions</td><td>Asthma and chronic lower respiratory disease</td><td>J41-J47</td></tr>
<tr><td>Sleep apnoea</td><td>G47.3</td></tr>
<tr><td>Other respiratory conditions</td><td>E84, J60-J70, J80-J86, J96.1, J98, P27, P75, Y55.6, Z43.0, Z93.0, Z94.2</td></tr>
<tr><td rowspan="5">Vision and hearing</td><td rowspan="2">Congenital eye conditions</td><td>Congenital cataract</td><td>Q12</td></tr>
<tr><td>Congenital eye conditions</td><td>Q10.4, Q10.7, Q11, Q13.0-Q13.4, Q13.8, Q13.9, Q14-Q16</td></tr>
<tr><td rowspan="2">Hearing loss</td><td>Glue ear, conductive hearing loss, and other chronic ear conditions</td><td>H60.2, H65.2-H65.4, H66.1-H66.3, H69.0, H70.1, H73.1, H74.0-H74.3, H75.0, H80, H81.0, H81.4, H83.0, H83.2, H90.0, H91, Z45.3</td></tr>
<tr><td>Sensorineural hearing loss</td><td>H90.3, H90.5, H90.6</td></tr>
<tr><td colspan="2">Non-congenital eye conditions</td><td>H05.1-H05.9, H13.3, H17, H18, H19.3, H19.8, H21, H26, H27, H28.0-H28.2, H31, H32.8, H33-H35, H40, H42.0, H43, H44, H47, H54.0- H54.2, H54.4, T85.2, T85.3, Z44.2</td></tr>
<tr><td colspan="3">Codes indicating non-specific chronic condition</td><td>R62, R63.3, Z43.1, Z51.2, Z51.5, Z54.2, Z75.5, Z93.1, Z99.3</td></tr>
</table>

Supplemental Table 3: The cumulative incidences of hospital-indicated chronic health conditions, by Down syndrome-status.

Category	Children with Down Syndrome				Children without Down Syndrome			
	**1 year**	**5 years**	**11 years**	**16 years**	**1 year**	**5 years**	**11 years**	**16 years**
First of any CHC (including nonspecific)	64.6 (63.7, 65.5)	81.2 (80.5, 82)	88 (87.3, 88.7)	90.1 (89.3, 90.8)	6.1 (6.1, 6.1)	12.4 (12.4, 12.5)	17.5 (17.5, 17.5)	21.2 (21.1, 21.2)
Multiple morbidity								
First category	63.6 (62.7, 64.5)	80.4 (79.7, 81.2)	87.4 (86.7, 88.1)	89.7 (88.9, 90.5)	5.4 (5.4, 5.4)	11.6 (11.6, 11.7)	16.7 (16.7, 16.8)	20.5 (20.4, 20.5)
Second category	30.3 (29.4, 31.1)	53.2 (52.2, 54.1)	66.1 (65.1, 67.2)	72.5 (71.3, 73.7)	1.0 (1.0, 1.0)	2.6 (2.6, 2.6)	4.3 (4.3, 4.3)	6.0 (6.0, 6.1)
Third category	13.4 (12.8, 14.1)	31.1 (30.2, 32.0)	44.6 (43.5, 45.7)	52.4 (51.0, 53.9)	0.4 (0.4, 0.4)	0.9 (0.9, 0.9)	1.5 (1.5, 1.5)	2.2 (2.2, 2.2)
Fourth category	5.4 (4.9, 5.8)	15.7 (15.0, 16.5)	25.9 (25.0, 27.0)	33.1 (31.7, 34.5)	0.1 (0.1, 0.1)	0.4 (0.4, 0.4)	0.6 (0.6, 0.6)	0.9 (0.9, 0.9)
Death (Kaplan-Meier estimate)	4.7 (4.3, 5.1)	6.2 (5.8, 6.7)	6.7 (6.2, 7.2)	7.0 (6.5, 7.5)	0.3 (0.3, 0.3)	0.4 (0.4, 0.4)	0.4 (0.4,0.4)	0.5 (0.5,0.5)
Autoimmune, endocrine, and metabolic conditions	7.0 (6.5, 7.5)	13.0 (12.3, 13.6)	20.5 (19.6, 21.4)	27.8 (26.4, 29.2)	0.5 (0.5, 0.5)	1.0 (1.0, 1.0)	1.5 (1.5, 1.5)	2.3 (2.3, 2.3)
Autoimmune conditions	5.3 (4.9, 5.8)	10.3 (9.7, 10.9)	16.2 (15.4, 17.1)	21.9 (20.6, 23.2)	0.4 (0.4, 0.4)	0.6 (0.6, 0.6)	0.7 (0.7, 0.7)	0.9 (0.9, 0.9)
Coeliac	1.1 (0.9, 1.3)	2.4 (2.1, 2.7)	4.5 (4.0, 5.0)	5.8 (5.2, 6.6)	0.3 (0.3, 0.3)	0.5 (0.5, 0.5)	0.6 (0.6, 0.6)	0.7 (0.7, 0.7)
Congenital hypothyroidism	2.7 (2.4, 3.0)	*[2]	*	4.5 (4.0, 4.9)	0.0 (0.0, 0.0)	*	*	0.1 (0.1, 0.1)
Acquired hypothyroidism	2.9 (2.6, 3.2)	7.1 (6.6, 7.6)	11.3 (10.6, 12)	15.8 (14.7, 17.1)	0.0 (0.0, 0.0)	0.0 (0.0, 0.0)	0.1 (0.1, 0.1)	0.1 (0.1, 0.1)
Hyperthyroidism	0.1 (0.0, 0.2)	**	1.2 (1.0, 1.5)	2.4 (1.9, 3.1)	0.0 (0.0, 0.0)	0.0 (0.0, 0.0)	0.0 (0.0, 0.0)	0.0 (0.0, 0.0)
Other metabolic and endocrine conditions	2.0 (1.8, 2.3)	3.5 (3.2, 3.9)	6.2 (5.6, 6.7)	10.0 (9.0, 11.1)	0.2 (0.2, 0.2)	0.5 (0.5, 0.5)	0.9 (0.9, 0.9)	1.5 (1.5, 1.6)
Diabetes	0.0 (0.0, 0.1)	*	0.9 (0.7, 1.2)	1.7 (1.3, 2.3)	0.0 (0.0, 0.0)	0.1 (0.1, 0.1)	0.2 (0.2, 0.3)	0.4 (0.4, 0.4)
Other endocrine and metabolic conditions	2 (1.7, 2.3)	3.2 (2.9, 3.6)	5.4 (4.9, 5.9)	8.7 (7.8, 9.8)	0.2 (0.2, 0.2)	0.4 (0.4, 0.4)	0.6 (0.6, 0.6)	1.1 (1.1, 1.2)
Cancers and blood disorders	5.6 (5.1, 6)	10 (9.4, 10.6)	11.8 (11.2, 12.5)	13.4 (12.5, 14.3)	0.3 (0.3, 0.4)	1.0 (1.0, 1.0)	1.5 (1.5, 1.5)	2.0 (2.0, 2.0)
Cancers	1.5 (1.3, 1.8)	2.7 (2.4, 3.1)	3.3 (3, 3.7)	3.7 (3.3, 4.2)	0.1 (0.1, 0.1)	0.2 (0.2, 0.2)	0.3 (0.3, 0.3)	0.4 (0.4, 0.4)
Acute lymphoblastic leukaemia	0.3 (0.2, 0.4)	0.9 (0.7, 1.1)	1.3 (1.1, 1.6)	1.5 (1.2, 1.8)	0.0 (0.0, 0.0)	0.0 (0.0, 0.0)	0.0 (0.0, 0.1)	0.1 (0.1, 0.1)
Acute myeloid leukaemia	0.3 (0.2, 0.4)	**[3]	**	1.1 (0.9, 1.3)	0.0 (0.0, 0.0)	0.0 (0.0, 0.0)	0.0 (0.0, 0.0)	0.0 (0.0, 0.0)
Other cancers	1.2 (1.0, 1.5)	2.4 (2.2, 2.8)	3.0 (2.7, 3.4)	3.3 (2.9, 3.8)	0.1 (0.0, 0.1)	0.2 (0.2, 0.2)	0.3 (0.3, 0.3)	0.4 (0.4, 0.4)
Blood disorders	4.8 (4.4, 5.2)	9.1 (8.6, 9.7)	10.9 (10.2, 11.5)	12.3 (11.5, 13.1)	0.3 (0.3, 0.3)	0.9 (0.9, 1)	1.4 (1.4, 1.4)	1.8 (1.8, 1.8)
Cardiovascular conditions	51.3 (50.3, 52.2)	57.9 (56.9, 58.8)	60.0 (59.0, 61.0)	61.1 (60.1, 62.1)	0.9 (0.9, 0.9)	1.2 (1.2, 1.2)	1.4 (1.4, 1.5)	1.8 (1.8, 1.8)
Severe congenital heart defects	49.6 (48.7, 50.6)	*	*	57.2 (56.2, 58.2)	0.6 (0.6, 0.6)	*	*	0.8 (0.8, 0.8)
Atrial septal defect	32.7 (31.8, 33.6)	*	*	40.9 (39.9, 41.9)	0.4 (0.4, 0.4)	*	*	0.5 (0.5, 0.5)
Atrioventricular septal defect	17.8 (17.1, 18.6)	*	*	20.5 (19.7, 21.3)	0.0 (0.0, 0.0)	*	*	0.0 (0.0, 0.0)
Ventricular septal defect	20.5 (19.8, 21.3)	*	*	25.0 (24.1, 25.9)	0.3 (0.3, 0.3)	*	*	0.3 (0.3, 0.3)
Tetralogy of Fallot	2.4 (2.1, 2.7)	*	*	2.7 (2.4, 3.0)	0.0 (0.0, 0.0)	*	*	0.0 (0.0, 0.0)
Other severe congenital heart defect	3.6 (3.2, 3.9)	*	*	5.4 (4.8, 6.0)	0.2 (0.2, 0.2)	*	*	0.2 (0.2, 0.2)
Non-congenital cardiovascular disease	19.4 (18.7, 20.2)	25.1 (24.3, 26)	27.7 (26.8, 28.6)	29.8 (28.8, 30.9)	0.5 (0.5, 0.5)	0.7 (0.7, 0.7)	0.9 (0.9, 1.0)	1.3 (1.3, 1.3)
Other cardiovascular disease	16.7 (16.1, 17.5)	22.3 (21.6, 23.2)	25.1 (24.2, 25.9)	27.4 (26.4, 28.5)	0.4 (0.4, 0.4)	0.7 (0.7, 0.7)	0.9 (0.9, 0.9)	1.2 (1.2, 1.3)
Pulmonary vascular disease	6.3 (5.9, 6.8)	7.9 (7.4, 8.4)	**	8.5 (8.0, 9.1)	0.0 (0.0, 0.0)	0.1 (0.1, 0.1)	0.1 (0.1, 0.1)	0.1 (0.1, 0.1)
Central nervous system (CNS) conditions	10.4 (9.9, 11)	16.2 (15.5, 16.9)	21.2 (20.4, 22.1)	25.0 (23.8, 26.2)	1.4 (1.4, 1.4)	2.3 (2.3, 2.3)	3.0 (3.0, 3.1)	3.9 (3.9, 3.9)
Congenital and perinatal anomalies of the CNS	6.6 (6.2, 7.1)	*	*	9.9 (9.2, 10.6)	0.9 (0.9, 0.9)	*	*	1.3 (1.3, 1.3)
Anomalies of the CNS	4.9 (4.5, 5.4)	*	*	8.3 (7.6, 9.0)	0.3 (0.3, 0.3)	*	*	0.7 (0.7, 0.7)
Perinatal conditions	2.1 (1.8, 2.3)	***[4]	***	***	0.6 (0.6, 0.6)	***	***	***
Non-congenital neurological conditions	4.8 (4.4, 5.2)	9.9 (9.4, 10.5)	15.1 (14.3, 15.9)	18.8 (17.7, 19.9)	0.7 (0.7, 0.7)	1.6 (1.5, 1.6)	2.2 (2.2, 2.2)	3.0 (3.0, 3.1)
Epilepsy	3.0 (2.7, 3.3)	5.2 (4.8, 5.7)	6.5 (6.0, 7.0)	7.4 (6.8, 8.1)	0.3 (0.3, 0.3)	0.9 (0.9, 0.9)	1.3 (1.2, 1.3)	1.5 (1.5, 1.6)

[2] Single asterisks (*) replace cumulative incidences for congenital conditions recorded by ages 5 and 11 years.
[3] Double asterisks (**) replace cumulative incidences representing changes in case counts between 0 and 10.
[4] Triple asterisks (***) replace cumulative incidences for perinatal conditions recorded after the age of 1 year.

Category	Children with Down Syndrome				Children without Down Syndrome			
Other neurological conditions	2.4 (2.1, 2.7)	6.2 (5.8, 6.7)	10.8 (10.1, 11.5)	14.0 (13.0, 15.1)	0.4 (0.4, 0.4)	0.8 (0.8, 0.8)	1.2 (1.2, 1.2)	1.8 (1.8, 1.9)
	**1 year**	**5 years**	**11 years**	**16 years**	**1 year**	**5 years**	**11 years**	**16 years**
Digestive, renal, and genitourinary conditions	17.1 (16.4, 17.9)	23.5 (22.7, 24.4)	26.4 (25.5, 27.3)	28.9 (27.8, 30)	2.0 (2.0, 2.0)	3.2 (3.2, 3.2)	3.9 (3.9, 4.0)	4.8 (4.7, 4.8)
Congenital digestive, renal, and GU anomalies	11.3 (10.8, 12)	*	*	14.0 (13.3, 14.8)	1.0 (1.0, 1.0)	*	*	1.3 (1.3, 1.3)
Upper gastro-intestinal anomalies	4.6 (4.2, 5.0)	*	*	5.0 (4.6, 5.4)	0.1 (0.1, 0.1)	*	*	0.1 (0.1, 0.1)
Lower gastro-intestinal anomalies	2.7 (2.4, 3.0)	*	*	2.9 (2.6, 3.2)	0.1 (0.1, 0.1)	*	*	0.1 (0.1, 0.1)
Other gastro-intestinal anomalies	2.4 (2.1, 2.7)	*	*	3.8 (3.4, 4.2)	0.2 (0.2, 0.2)	*	*	0.3 (0.3, 0.3)
Kidney and urinary tract anomalies	2.9 (2.6, 3.2)	*	*	3.7 (3.3, 4.1)	0.4 (0.4, 0.4)	*	*	0.6 (0.6, 0.6)
Genital anomalies	0.7 (0.6, 0.9)	*	*	1.5 (1.2, 2.0)	0.3 (0.3, 0.3)	*	*	0.5 (0.5, 0.5)
Non-congenital digestive, renal, and GU	10.0 (9.5, 10.6)	16.4 (15.7, 17.2)	19.7 (18.9, 20.5)	22.5 (21.4, 23.7)	1.2 (1.2, 1.2)	2.3 (2.3, 2.3)	3.0 (3.0, 3.0)	3.8 (3.8, 3.8)
Digestive conditions	7.2 (6.7, 7.7)	12.9 (12.2, 13.5)	15.5 (14.7, 16.2)	17.8 (16.7, 18.8)	0.9 (0.9, 0.9)	1.9 (1.9, 1.9)	2.3 (2.3, 2.3)	2.8 (2.8, 2.9)
Renal and GU conditions	4.0 (3.6, 4.4)	6.0 (5.5, 6.5)	7.8 (7.2, 8.3)	9.4 (8.7, 10.3)	0.3 (0.3, 0.3)	0.5 (0.5, 0.5)	0.9 (0.9, 0.9)	1.2 (1.2, 1.2)
Developmental and behavioural conditions	1.7 (1.4, 1.9)	10.4 (9.8, 11.1)	23.6 (22.6, 24.6)	34.4 (32.9, 36.0)	0.0 (0.0, 0.0)	0.8 (0.8, 0.8)	1.8 (1.8, 1.8)	2.8 (2.8, 2.8)
Autism spectrum disorders	--[5]	0.9 (0.7, 1.1)	4.7 (4.2, 5.3)	9.2 (8.2, 10.4)	--	0.2 (0.2, 0.2)	0.7 (0.7, 0.7)	1.3 (1.3, 1.3)
Developmental and intellectual disabilities	1.7 (1.4, 1.9)	9.7 (9.1, 10.3)	20.5 (19.6, 21.5)	29.7 (28.2, 31.3)	0.0 (0.0, 0.0)	0.6 (0.6, 0.6)	1.0 (1.0, 1.1)	1.3 (1.3, 1.3)
Behavioural and emotional disorders	--	0.3 (0.2, 0.5)	1.9 (1.5, 2.2)	4.1 (3.4, 4.9)	--	0.1 (0.1, 0.1)	0.4 (0.4, 0.4)	1.0 (0.9, 1.0)
Musculoskeletal and skin conditions	4.1 (3.7, 4.5)	6.5 (6.0, 7.0)	10.1 (9.4, 10.8)	14.0 (12.9, 15.2)	0.5 (0.5, 0.5)	1.0 (1.0, 1.0)	1.5 (1.5, 1.5)	2.2 (2.2, 2.2)
Congenital musculoskeletal and skin anomalies	3.3 (3.0, 3.7)	*	*	5.5 (5.0, 6.1)	0.4 (0.4, 0.4)	*	*	0.8 (0.8, 0.8)
Non-congenital musculoskeletal and skin	1.0 (0.8, 1.2)	3.0 (2.7, 3.4)	6.2 (5.7, 6.8)	10.3 (9.2, 11.4)	0.2 (0.2, 0.2)	0.4 (0.4, 0.4)	0.9 (0.9, 0.9)	1.6 (1.6, 1.6)
Inflammatory arthritis	**	**	0.3 (0.2, 0.5)	0.6 (0.4, 1.1)	0.0 (0.0, 0.0)	0.0 (0.0, 0.0)	0.1 (0.1, 0.1)	0.1 (0.1, 0.1)
Chronic skin disorders	0.6 (0.5, 0.8)	1.5 (1.3, 1.8)	2.4 (2.1, 2.8)	3.2 (2.7, 3.7)	0.1 (0.1, 0.1)	0.2 (0.2, 0.2)	0.3 (0.3, 0.3)	0.5 (0.5, 0.5)
Musculoskeletal and connective tissue	0.4 (0.3, 0.5)	1.5 (1.2, 1.7)	3.7 (3.3, 4.2)	6.1 (5.3, 7.0)	0.0 (0.0, 0.0)	0.2 (0.2, 0.2)	0.5 (0.5, 0.5)	1.0 (1.0, 1.0)
Scoliosis	**	0.2 (0.1, 0.3)	0.5 (0.3, 0.7)	1.8 (1.3, 2.5)	0.0 (0.0, 0.0)	0.0 (0.0, 0.0)	0.1 (0.1, 0.1)	0.1 (0.1, 0.2)
Respiratory conditions	13.8 (13.2, 14.5)	32.2 (31.3, 33.1)	43.6 (42.5, 44.6)	47.7 (46.5, 49.0)	0.9 (0.9, 0.9)	3.6 (3.6, 3.6)	6.0 (6.0, 6.1)	7.5 (7.5, 7.5)
Congenital respiratory anomalies	5.8 (5.3, 6.2)	*	*	9.7 (9.0, 10.3)	0.4 (0.4, 0.4)	*	*	0.5 (0.5, 0.5)
Non-congenital respiratory conditions	10.3 (9.8, 10.9)	29.2 (28.3, 30.1)	41.0 (40.0, 42.1)	45.3 (44.0, 46.6)	0.5 (0.5, 0.5)	3.2 (3.2, 3.2)	5.7 (5.7, 5.7)	7.1 (7.1, 7.1)
Sleep apnoea	1.8 (1.6, 2.1)	17.1 (16.4, 17.9)	28.0 (27.0, 29.1)	32.2 (31.0, 33.5)	0.0 (0.0, 0.0)	0.8 (0.8, 0.8)	1.3 (1.3, 1.3)	1.4 (1.4, 1.4)
Asthma and lower respiratory disease	0.5 (0.4, 0.7)	5.6 (5.2, 6.1)	10.4 (9.7, 11.1)	12.6 (11.7, 13.6)	0.1 (0.1, 0.1)	2.0 (1.9, 2.0)	3.9 (3.9, 4.0)	5.3 (5.3, 5.3)
Other respiratory conditions	8.7 (8.2, 9.2)	13.3 (12.6, 14)	14.9 (14.2, 15.6)	15.8 (15.0, 16.7)	0.4 (0.4, 0.4)	0.7 (0.7, 0.7)	0.8 (0.8, 0.8)	0.9 (0.9, 0.9)
Vision and hearing	4.2 (3.8, 4.6)	23.4 (22.6, 24.3)	33.5 (32.5, 34.6)	37.0 (35.7, 38.3)	0.3 (0.3, 0.3)	1.7 (1.7, 1.7)	2.9 (2.9, 2.9)	3.2 (3.2, 3.2)
Congenital eye conditions	1.4 (1.2, 1.7)	*	*	2.4 (2.1, 2.8)	0.1 (0.1, 0.1)	*	*	0.1 (0.1, 0.1)
Non-congenital eye conditions	1.4 (1.2, 1.6)	2.6 (2.3, 3.0)	3.7 (3.4, 4.2)	4.9 (4.3, 5.7)	0.2 (0.2, 0.2)	0.3 (0.3, 0.3)	0.4 (0.4, 0.4)	0.5 (0.5, 0.5)
Hearing loss	1.9 (1.6, 2.1)	21.0 (20.2, 21.8)	30.7 (29.7, 31.7)	33.7 (32.5, 35.0)	0.1 (0.1, 0.1)	1.4 (1.4, 1.4)	2.5 (2.5, 2.5)	2.6 (2.6, 2.7)
Conductive hearing loss and glue ear	1.6 (1.4, 1.9)	20.5 (19.7, 21.4)	30.1 (29.1, 31.2)	33.3 (32.0, 34.5)	0.1 (0.1, 0.1)	1.3 (1.3, 1.4)	2.4 (2.4, 2.5)	2.6 (2.6, 2.6)
Sensorineural hearing loss	0.3 (0.2, 0.5)	1.5 (1.2, 1.7)	2.4 (2.1, 2.8)	3.2 (2.7, 3.8)	0.0 (0.0, 0.0)	0.1 (0.1, 0.1)	0.1 (0.1, 0.1)	0.1 (0.1, 0.1)
Non-specific chronic conditions	14.3 (13.6, 15.0)	23.0 (22.2, 23.8)	26.8 (25.9, 27.7)	28.8 (27.7, 29.9)	1.2 (1.2, 1.2)	1.9 (1.9, 1.9)	2.2 (2.2, 2.2)	2.3 (2.3, 2.3)

[5] Double dashes (--) replace cumulative incidences for autism spectrum disorders and behavioural emotional disorders by age 1.

Supplemental Figure 1: The cumulative incidence of congenital conditions indicated by age 16 years among children with Down syndrome (DS)

Congenital chronic health conditions among children with DS

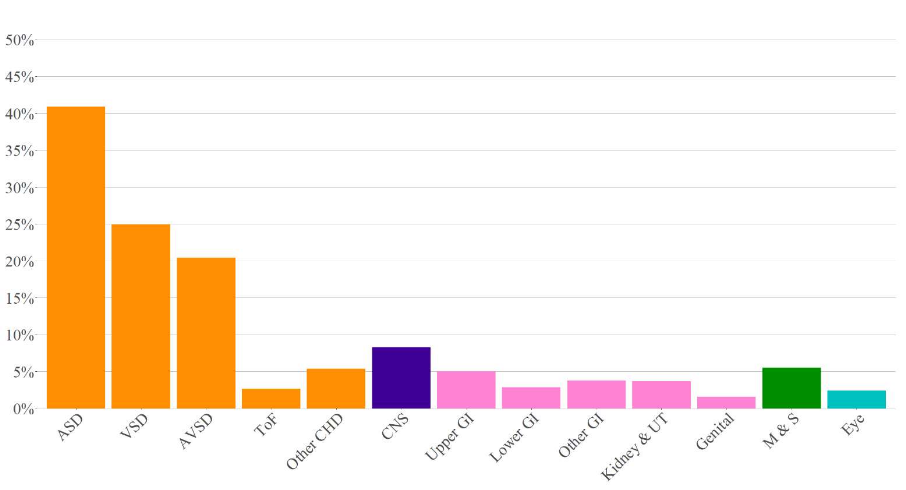

Congenital condition categories
Cardiovascular
Central nervous system
Digestive, renal, & genitourinary
Musculoskeletal & skin
Vision and hearing

Abbreviations	
ASD	Atrial septal defects
VSD	Ventricular septal defects
AVSD	Atrioventricular septal defect
ToF	Tetralogy of Fallot
CHD	Congenital heart defect
CNS	Central nervous system
GI	Gastrointestinal
UT	Urinary tract
M & S	Musculoskeletal & skin

We depicted the cumulative incidence by 16 years of congenital anomalies in the DS group using a histogram because the time-to-indication for anomalies represents delayed recording, rather than the development of anomalies.

Supplemental Figure 2: The cumulative incidence of mortality, by Down syndrome (DS)-status

Mortality

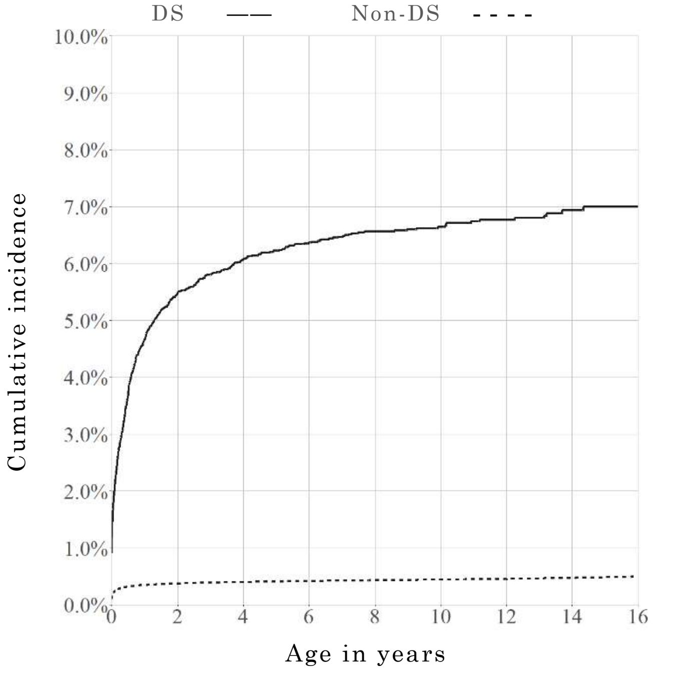

Supplemental Figure 3: Sensitivity analysis showing the cumulative incidence of any recorded chronic health condition, by birth year, by Down syndrome (DS)-status

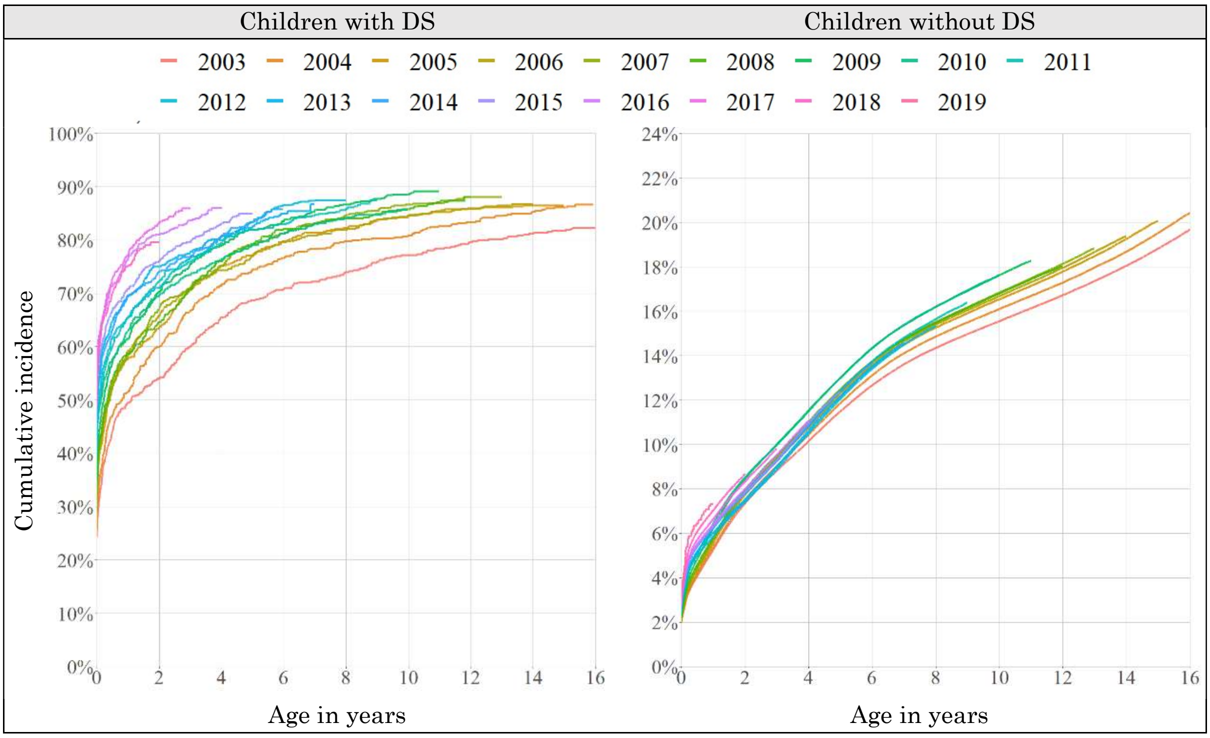

Supplemental Figure 4: Coding depth, represented as the average count of ICD-10 codes recorded per hospital episode for children with Down syndrome (DS) and children without DS who had at least one chronic health condition recorded by age 16.

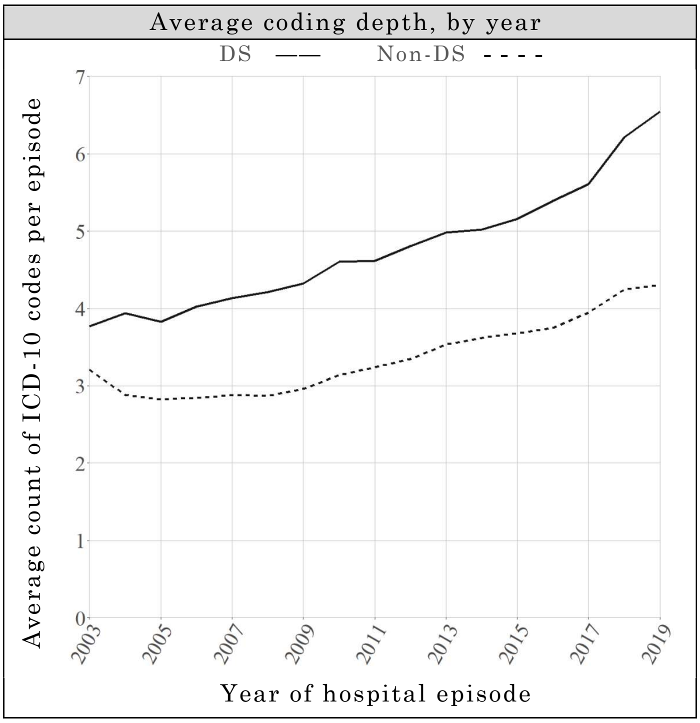

Supplemental Table 4: Comparison between chronic health condition cumulative incidences in the HES 2003-2019 Down syndrome cohort and period prevalences presented in other studies

**Paper**	McKenna, C.(3)		Fitzgerald, et al.(4)		Current study	
**Cohort**	Children in England with DS		Population of children with DS born in Western Australia		Liveborn, singleton children with DS born in English hospitals	
**Data sources**	Hospital Episode Statistics (HES), National Cancer Registration and Analysis Service, Clinical Practice Research Data Link, Office of National Statistics (ONS)		Western Australian Intellectual Disability database; Hospital Morbidity Data System		HES, ONS	
N	1,340		405		10,621	
**Location**	England		Western Australia		England	
**Birth dates**	NA		1 January 1983-31 December 1999		1 January 2003-1 March 2019	
**Follow-up period**	1990-31 December 2017 for cancers, April 1997 – 31 December 2017 for HES		1 January 1983-31 December 2004		1 January 2003-1 March 2020	
**Follow-up ages**	0-18 years, median: 3.9 years.		0.21-21.86 years, median: 12.59 years.		0-16 years, median: 8.3 years.	
**Reported Measures**	Period prevalence		Period prevalence		Cumulative incidence and confidence interval up to age 16	
Death	Death	3.1%	Death	8.9%	Death	7.0 (6.5, 7.5)
Autoimmune, endocrine, and metabolic			Endocrine/ metabolic/immune	8.4%	Any	27.8 (26.4, 29.2)
	Hyperthyroid	2.5% (1.8-3.5%)			Hyperthyroid	2.4 (1.9, 3.1)
	Hypothyroid	15.8% (14.0-17.8%)			Acquired hypothyroid	15.8 (14.7, 17.1)
	Diabetes mellitus	2.8% (2.0-3.8%)			Diabetes	1.7 (1.3, 2.3)
Cancers	Leukaemia	2.2% (1.5-3.1%)	Leukaemia	4.0%	ALL	1.5 (1.2, 1.8)
	Lymphoma	0.2% (0.1%-0.7%)			AML	1.1 (0.9, 1.3)
Cardiovascular			Any	26.7%	Any	61.1 (60.1, 62.1)
	Congenital conditions	56.3% (53.6-58.9%)	Cardiac septum defects	22.7%	Severe CHD	57.2 (56.2, 58.2)
			Patent ductus arteriosus	3.5%		
Central nervous system			Any	4.9%	Any	25.0 (23.8, 26.2)
	Epilepsy	6.0% (4.8-7.3%)	Epilepsy	1.2%	Epilepsy	7.4 (6.8, 8.1)
			Non-febrile convulsions	1.2%		
Digestive, renal, and genitourinary (GU)	Coeliac	2.8% (2.1-3.9%)			Coeliac	5.8 (5.2, 6.6)
	Congenital gastrointestinal disease	4.7% (3.7-6.0%)	Congenital digestive abnormalities	6.9%	Congenital digestive, renal, and GU anomalies	14.0 (13.3, 14.8)
	Chronic kidney disease	1.1% (.7-1.9%)	Bladder	3.2%	Renal and GU conditions (non-congenital)	9.4 (8.7, 10.3)
Developmental and behavioural	Autism	6.3% (5.2-7.8%)			Autism spectrum disorders	9.2 (8.2, 10.4)
	Anxiety/ depression	1.9% (1.3-2.8%)			Behavioural and emotional disorders	4.1 (3.4, 4.9)
Musculoskeletal and skin	Atlantoaxial instability	0.8% (0.5-1.5%)	Congenital musculoskeletal conditions	2.50%	Congenital anomalies	5.5 (5.0, 6.1)
	Arthritis (combined)	0.9% (0.5-1.6%)			Inflammatory arthritis	0.6 (0.4, 1.1)
Respiratory	Sleep disordered breathing	19.1% (17.1-21.3%)	Sleep apnoea	5.2%	Sleep apnoea	32.2 (31.0, 33.5)
			Asthma	6.9%	Asthma and chronic lower respiratory	12.6 (11.7, 13.6)
			Congenital anomalies	1.5%	Congenital anomalies	9.7 (9.0, 10.3)
Vision and hearing	Cataract	2.1% (1.5-3.0%)	Congenital eye anomalies	0.5%	Congenital eye conditions	2.4 (2.1, 2.8)
	Hearing impairment	23.5% (21.3-25.9%)	Otitis media	47.9%	Conductive hearing loss & glue ear	33.3 (32.0, 34.5)

References for Supplemental Material

1. European Surveillance of Congenital Anomalies (EUROCAT). Chapter 3.3 - EUROCAT Subgroups of Congenital Anomalies. In: EUROCAT Guide 15 [Internet]. [cited 2023 Oct 23]. Available from: https://eu-rd-platform.jrc.ec.europa.eu/system/files/public/eurocat/Guide_1.5_Chapter_3.3.pdf

2. Dolk H, Loane M, Garne E, European Surveillance of Congenital Anomalies (EUROCAT) Working Group. Congenital heart defects in Europe: prevalence and perinatal mortality, 2000 to 2005. Circulation. 2011 Mar 1;123(8):841–9.

3. McKenna C. The health and care of children with Down Syndrome [Internet] [MD (Res) thesis]. [London, England]: University College London, Great Ormond Street Institute of Child Health; 2021 [cited 2024 Jan 9]. Available from: https://discovery.ucl.ac.uk/id/eprint/10134480/1/McKenna_10134480_Thesis_sig_removed.pdf

4. Fitzgerald P, Leonard H, Pikora TJ, Bourke J, Hammond G. Hospital Admissions in Children with Down Syndrome: Experience of a Population-Based Cohort Followed from Birth. PLOS ONE. 2013 Aug 13;8(8):e70401.
